# Rengasvirus, a Circular Replication-Associated Protein-Encoding Single-Stranded DNA Virus-Related Genome That Is a Common Contaminant in Metagenomic Data

**DOI:** 10.1128/MRA.00273-21

**Published:** 2021-05-06

**Authors:** Emma L. Keeler, Louis J. Taylor, Arwa Abbas, Ronald G. Collman, Frederic D. Bushman

**Affiliations:** a Department of Microbiology, Perelman School of Medicine, University of Pennsylvania, Philadelphia, Pennsylvania, USA; b Department of Medicine, Pulmonary, Allergy and Critical Care Division, Perelman School of Medicine, University of Pennsylvania, Philadelphia, Pennsylvania, USA; DOE Joint Genome Institute

## Abstract

We report the genome of a circular replication-associated protein (Rep)-encoding segmented or satellite virus, which we have provisionally named rengasvirus. In metagenomic studies of virus-enriched fractions, rengasvirus was detected widely, including in reagent-negative controls. We thus report this genome to help others recognize a probable contaminating sequence.

## Abstract (precis)

We report the genome of a circular Rep-encoding segmented or satellite virus, which we have provisionally named rengasvirus. In metagenomic studies of virus-enriched fractions, rengasvirus was detected widely, including in reagent-negative controls. We thus report this genome to help others recognize a probable contaminating sequence.

## ANNOUNCEMENT

Here, we describe a circular replication-associated protein (Rep)-encoding single-stranded (CRESS) DNA virus-related genome discovered in metagenomic data from human subjects and also in negative controls, suggesting that it originates from laboratory reagents. The rengasviral (*rengas* = Finnish for “ring”) sequence was first detected in metagenomic sequence data from bronchoalveolar lavage (BAL) fluid samples from lung transplant recipients (1). Default parameters were used for all software except where otherwise indicated. Contig building, open reading frame (ORF) prediction, and mapping of reads to contigs were performed using the Sunbeam pipeline version 2.1 (2); candidate CRESS viruses were identified by screening against vFam models for CRESS viral Reps using HMMER version 3 (3, 4). We confirmed the circularity of these sequences using PCR by amplifying around the DNA circles. For this, we used divergently oriented “back-to-back” primer pairs and recovered product bands of genome length. This was repeated with two primer sets binding to different locations on the circular DNA (set A forward [Fwd], GGCAGATCTAGATCACTACTCTGGAC; set A reverse [Rev], GCCAATGCGGGAGTAAATAGCTTG; set B Fwd, CCCTATCACTCTATAACATAACAAATGTCATTAGG; set B Rev, GGGTAATACTGATCCTATCACTCCTTTATAAC). We identified 105× coverage of the PCR-confirmed rengasvirus full-genome sequence in the sample in which we initially identified the rengasvirus sequence (SRA run accession number SRR5826708).

**FIG 1:**
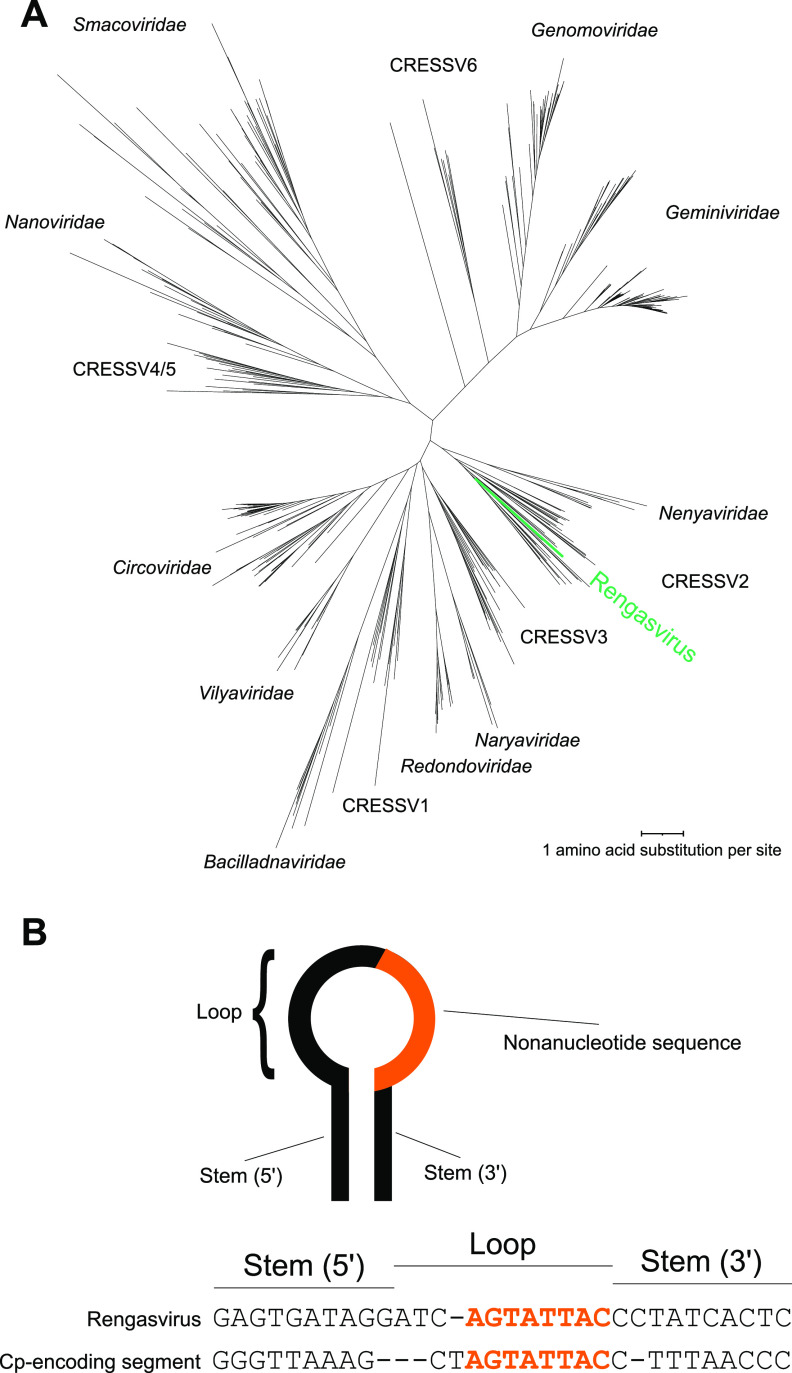
(A) Phylogenetic tree of CRESS virus Rep amino acid sequences. The rengasvirus Rep is shown in green. Rep amino acid sequences were aligned using MUSCLE version 3.8; trees were constructed using RaxML version 8.2 and visualized using iTOL version 6. (B) Comparison between rengasvirus DNA stem-loop and that of a circular DNA encoding capsid found in one rengasvirus-positive sample.

The rengasvirus genome is a 1,045-bp DNA sequence containing a single ORF encoding the Rep with a GC content of 49.7% (GenBank accession number MW559600). Based on BLASTp searches, the closest reported Rep amino acid sequences were from a circular DNA molecule from a glacial ice core (QGF19362.1), a CRESS virus helicase (AWW06123.1), and a dragonfly larva-associated circular virus (ALE29688.1), with sequence identities of 51.49%, 42.91%, and 41.11%, respectively (online search, February 2021). A maximum-likelihood phylogenetic tree of Rep placed rengasvirus as a member of the CRESSV2 viral cluster (Fig. 1A) (5).

To investigate the prevalence of rengasvirus sequences, we interrogated publicly available metagenomic data sets for homologous sequences generated by our lab and by other groups. Alignments were performed using the hisss pipeline (https://github.com/louiejtaylor/hisss), described in reference 6, which uses grabseqs and sra-tools to access public metagenomic data, Bowtie 2 (option, –very-sensitive-local) to align reads to target genomes, and ggplot2 (R version 3.2.3) (7–11). A positive rengasvirus hit in a metagenomic sample was defined as reads aligning to ≥25% of the viral genome; we discussed the rationale for this cutoff for CRESS virus genomes in a previous publication (6). Of the 40 data sets and 3,568 samples queried for sequence homology to the rengasvirus genome, positive hits were detected in 6 data sets, with percentages of positive samples ranging from 0.70% to 10.9% of samples (Table 1). We identified hits to the rengasvirus genome in various control samples from two different in-house studies, including two buffer-negative controls performed using the All Prep extraction kit (SRA numbers SRR6316280 and SRR6316219) (1) and one water extraction blank using the UltraSens virus kit (SRR7430813) (both kits from Qiagen, Valencia, CA) (12). Few public data sets include sequenced negative controls, precluding a detailed analysis of the origin of this putative genome or segment. However, circular DNAs have previously been identified as contaminants in nucleic extraction kit columns (13), representing a potential source for rengasvirus DNA in negative-control samples.

**TABLE 1:** Metadata associated with rengasvirus-positive metagenome samples (≥25% coverage)

BioProject no.	Organism	Body site	Sample	Disease state	Location	Positive samples (%)
PRJNA390659	Homo sapiens; controls	Lung	BAL fluid; buffer	Lung transplant	United States	10.9
PRJNA327423	Homo sapiens	Gut; oral	Stool; saliva	None	United States	1.04
PRJNA385126	Homo sapiens	Gut	Stool	None	Ireland	2.50
PRJNA275568	Homo sapiens	Gut	Stool	Islet autoimmunity	Finland	5.21
PRJEB9524	Homo sapiens	Gut	Stool	HIV	Uganda	1.52
PRJNA407341	Homo sapiens	Gut	Stool	IBD^*a*^	Ireland	0.70

a IBD, inflammatory bowel disease.

The rengasvirus genome described encodes only a Rep, raising the question of how it becomes encapsidated in viral particles. In one BAL fluid sample containing rengasvirus (SRA number SRR5826708), we also identified another circular DNA of 933 bases in length encoding a capsid protein with a GC content of 41.3% (GenBank accession number MW559599). We identified this sequence from metagenomic contigs using a method similar to the initial rengasvirus detection method (described above), except for using hidden Markov models (HMMs) based on viral capsid instead of Reps from vFam (3). For this molecule, we also confirmed circularity using whole-genome PCR with two sets of back-to-back primers as described above (set A Fwd, GCCTCACTTAAATAGATGTTAAGGTATGCAATG; set A Rev, GGCAAGTACTGGTACTGCACC; set B Fwd, GCCATAAGCATTCCGCGTG; set B Rev, GGCGAAGAGGAAGAGGAAGATG). This sequence also contained a DNA stem-loop with some resemblance to that of the rengasvirus Rep-encoding DNA (Fig. 1B). Thus, the two molecules together might comprise a bipartite genome. It is also possible that rengasvirus is a satellite virus relying on capsid and other functions produced by another unknown virus.

In summary, our results indicate that rengasvirus sequences are a common laboratory contaminant and provide an alignment target that can be used for quality control in future metagenome studies.

### Data availability.

The sequences described above been deposited in GenBank under the accession numbers MW559599 and MW559600. The sequence data set in which both sequences were originally identified is available under BioProject number PRJNA390659.

## References

[1] Abbas A, Diamond JM, Chehoud C, Chang B, Kotzin JJ, Young JC, Imai I, Haas AR, Cantu E, Lederer DJ, Meyer KC, Milewski RK, Olthoff KM, Shaked A, Christie JD, Bushman FD, Collman RG. 2017. The perioperative lung transplant virome: torque teno viruses are elevated in donor lungs and show divergent dynamics in primary graft dysfunction. Am J Transplant 17:1313–1324. doi:10.1111/ajt.14076. 27731934 PMC5389935

[2] Clarke EL, Taylor LJ, Zhao C, Connell J, Lee J-J, Fett B, Bushman FD, Bittinger K. 2019. Sunbeam: an extensible pipeline for analyzing metagenomic sequencing experiments. Microbiome 7:2–13. doi:10.1186/s40168-019-0658-x. 30902113 PMC6429786

[3] Skewes-Cox P, Sharpton TJ, Pollard KS, DeRisi JL. 2014. Profile hidden Markov models for the detection of viruses within metagenomic sequence data. PLoS One 9:e105067. doi:10.1371/journal.pone.0105067. 25140992 PMC4139300

[4] Finn RD, Clements J, Eddy SR. 2011. HMMER Web server: interactive sequence similarity searching. Nucleic Acids Res 39:29–37. doi:10.1093/nar/gkr367. PMC3125773 21593126

[5] Krupovic M, Varsani A, Kazlauskas D, Breitbart M, Delwart E, Rosario K, Yutin N, Wolf YI, Harrach B, Zerbini FM, Dolja VV, Kuhn JH, Koonin EV. 2020. Cressdnaviricota: a virus phylum unifying seven families of Rep-encoding viruses with single-stranded, circular DNA genomes. J Virol 94:1–4. doi:10.1128/JVI.00582-20. PMC7307096 32269128

[6] Abbas A, Taylor LJ, Dothard MI, Leiby JS, Fitzgerald AS, Khatib LA, Collman RG, Bushman FD. 2019. Redondoviridae, a family of small, circular DNA viruses of the human oro-respiratory tract associated with periodontitis and critical illness. Cell Host Microbe 25:719–729. doi:10.1016/j.chom.2019.04.001. 31071295 PMC6510254

[7] Taylor LJ, Abbas A, Bushman FD. 2020. grabseqs: simple downloading of reads and metadata from multiple next-generation sequencing data repositories. Bioinformatics 36:3607–3609. doi:10.1093/bioinformatics/btaa167. 32154830 PMC7267817

[8] Leinonen R, Sugawara H, Shumway M. 2011. The sequence read archive. Nucleic Acids Res 39:D19–D21. doi:10.1093/nar/gkq1019. 21062823 PMC3013647

[9] Langmead B, Salzberg SL. 2012. Fast gapped-read alignment with Bowtie 2. Nat Methods 9:357–359. doi:10.1038/nmeth.1923. 22388286 PMC3322381

[10] Wickham H. 2016. ggplot2: elegant graphics for data analysis. Springer, New York, NY.

[11] Ihaka R, Gentleman R. 2012. R: a language for data analysis and graphics 5. J Comput Graph Stat 5:299–314.

[12] Clarke EL, Connell AJ, Six E, Kadry NA, Abbas AA, Hwang Y, Everett JK, Hofstaedter CE, Marsh R, Armant M, Kelsen J, Notarangelo LD, Collman RG, Hacein-Bey-Abina S, Kohn DB, Cavazzana M, Fischer A, Williams DA, Pai SY, Bushman FD. 2018. T cell dynamics and response of the microbiota after gene therapy to treat X-linked severe combined immunodeficiency. Genome Med 10:70. doi:10.1186/s13073-018-0580-z. 30261899 PMC6161392

[13] Naccache SN, Greninger AL, Lee D, Coffey LL, Phan T, Rein-Weston A, Aronsohn A, Hackett J, Delwart EL, Chiu CY. 2013. The perils of pathogen discovery: origin of a novel parvovirus-like hybrid genome traced to nucleic acid extraction spin columns. J Virol 87:11966–11977. doi:10.1128/JVI.02323-13. 24027301 PMC3807889

